# Echocardiography in acute rheumatic fever

**DOI:** 10.4103/0974-2069.52812

**Published:** 2009

**Authors:** S Ramakrishnan

**Affiliations:** Department of Cardiology, All India Institute of Medical Sciences, New Delhi, India

## INTRODUCTION

“It has become appallingly obvious that our technology has exceeded our humanity”

– Albert Einstein.

Rheumatic heart disease (RHD) continues to remain a major health concern across the globe. It is estimated that a minimum of 15.6 million people have RHD in the world, with 2,82,000 new cases being added each year, and is estimated to cause 2,33,000 deaths each year.[1–3] RHD results in enormous disease burden translating into huge economic and social losses in India. Classical rheumatic fever (RF) is still encountered across the country. The diagnosis of RF remains clinical. Valvulitis is *sine qua* non of acute carditis in RF and no investigation is better than echocardiography in its assessment. Echocardiography along with Doppler assessment gives excellent details of the structural and functional abnormalities in acute RF. Yet, echocardiography is not included as a criterion in the diagnosis of RF. No other indication for echocardiography has undergone such a rigorous scrutiny. In this review, a critical appraisal of the role of echocardiography in the diagnosis of RF is presented.

## ECHOCARDIOGRAPHIC FEATURES OF RHEUMATIC CARDITIS

The echocardiographic features of acute rheumatic carditis are summarized in Table 1. Assessment of valvular regurgitation has to be accurate as there is a continuum between physiological and pathological regurgitation.[3,4] The characteristics of a physiological regurgitation include localized jet at the region immediately below or above the plane of valve leaflets (or within 1.0 cm), short signals and a smaller maximum regurgitant area.[3,4] The reported prevalence of physiological valvular regurgitation in normal people is mitral regurgitation in 2.4-45%,[5,6] aortic regurgitation in 0-33%,[5,6] tricuspid regurgitation in 6.3- 95%[6,7] and pulmonary regurgitation in 21.9-92%.[6,8] Hence, definite criteria for pathological regurgitation have been proposed [Table 1] and are widely used.[3] Rheumatic nodules (beaded appearance of leaflets) are found in nearly 40-50% of the patients with acute carditis and are shown to disappear following treatment of carditis.[9,10] Nodules may be more useful for diagnosing recurrence of RF among patients with established RHD, but the sensitivity and specificity of these nodules is not known. The utility of any other structural abnormality for the echocardiographic diagnosis of acute rheumatic carditis in the absence of pathological valvular regurgitation is not well established. A recent study had used a composite score of 8 echocardiographic parameters and concluded that a score of 6 out of 16 identifies echocardiographic carditis precisely.[11] However, the majority of the patients with carditis in that study had pathological mitral or aortic regurgitation.

**Table 1:** Echocardiographic features of rheumatic carditis

Valvular regurgitation (WHO suggested)[3]
A regurgitant jet >1 cm in length
A regurgitant jet in at least two planes
A mosaic color jet with a peak velocity >2.5 m/s
Jet persists throughout systole (mitral valve) and diastole (aortic valve)
Leaflet
Prolapse
Coaptation failure
Thickening (>4 mm)
Reduced mobility
Nodules
Annular dilatation
Chordal elongation/rupture
Increased echogenicity of subvalvular apparatus
Pericardial effusion
Ventricular dilatation and dysfunction (almost always with significant regurgitation)

## SUBCLINICAL CARDITIS

A significant number of patients with suspected acute rheumatic carditis have no clinical murmurs but have documented regurgitation on echocardiography. Thus, a new category of “subclinical carditis,, “echocarditis” or “asymptomatic carditis” has emerged. Subclinical carditis is relatively common in RF.[12] The reported prevalence of subclinical carditis in RF ranges from 0 to 53% [Table 2]. The probable causes for widely varying estimates include population studied, expertise of physician, echocardiographic criteria used and referral bias (secondary/tertiary setting). A metaanalysis of various studies on subclinical carditis reported a weighted pooled prevalence of 16.8% (95% confidence interval [CI] 11.9-21.6). With the application of the World Health Organization echocardiographic criteria, the prevalence of subclinical carditis increased slightly to 18.1% (95% CI 11.1-25.2).[12] These estimates are based on nearly 20 studies that included more than 1700 RF cases from areas of the globe with high prevalence of RHD, except sub-Saharan Africa.

**Table 2:** Studies of subclinical carditis using WHO criteria in rheumatic fever

Country	Year	Number of cases	Clinical carditis (%)	Subclinical carditis (%)	No carditis (%)
Brazil[13]	2008	56	27 (48.2)	11 (19.6)	18 (32.1)
Nepal[14]	2007	51	40 (78.4)	5 (9.8)	6 (11.8)
Thailand[15]	2004	44	17 (39)	3 (7)	24 (55)
Turkey[16]	2003	104	51 (49)	23 (22)	30 (29)
Brazil[17]	2003	40	28 (70)	2 (5)	10 (25)
Chile[18]	2001	35	15 (43)	11 (31)	9 (25)
New Zealand[19]	2000	59	35 (59)	8 (14)	16 (27)
India[20]	2000	163	110 (67)	11 (7)	42 (26)
Turkey[21]	1999	22	5 (23)	9 (41)	8 (36)
USA[22]	1997	27	21 (78)	2 (7)	4 (15)
Qatar[23]	1992	19	8 (42)	10 (53)	1 (5)
Qatar[24]	1989	22	12 (55)	8 (36)	2 (9)
Brazil*[25]	1999	22	8 (36)	5 (23)	9 (41)
France*[26]	1995	100	50 (50)	30 (30)	20 (20)
India*[27]	1995	63	35 (56)	0 (0)	28 (44)
New Zealand*[28]	1994	34	15 (44)	5 (15)	14 (41)

* Incomplete use of WHO criteria, Modified from Tubridy–Clark M and Carapetis JR 2007[12]

The outcome of patients with subclinical carditis is less well established. The metaanalysis reported a weighted pooled prevalence of persistence or deterioration of subclinical carditis at 3-23 months after diagnosis in 44.7% (95% CI 19.3-70.2).[12] These studies confirm that subclinical carditis persists in similar proportions to that of mild clinical carditis. However, the follow-up was not uniform and included only 99 patients among 11 studies. More importantly, the degree of worsening, details of secondary prophylaxis and proportion of patients in whom murmurs appeared on follow-up are not known.

## INCLUSION OF ECHOCARDIOGRAPHY AS A “JONES CRITERION” – THE CONS

For echocardiography to be included as a diagnostic criterion, three things need to be established. Firstly, the incidence of subclinical carditis should be significant. Secondly, the outcome of subclinical carditis should not be benign and thirdly, treatment or prophylaxis should alter the outcome. Even though subclinical carditis is shown to be relatively frequent, the outcome is not well established. It is not known whether subclinical carditis follows the same evolution as audible valvular lesions. Studies of long-term follow-up have most likely included most of the patients with subclinical carditis in the no-carditis group, which is shown to have uniformly good prognosis. Additional diagnosis of carditis by echocardiography does not alter the acute treatment as only symptomatic treatment is needed in patients with mild valvular lesions. However, such patients may need additional secondary prophylaxis because the duration of prophylaxis is determined by the presence or absence of carditis.[29] Hence, it may be argued that it is wiser to perform an echocardiography before stopping prophylaxis in countries with limited resources.

Fever alone may produce pathological regurgitation that disappears with the offset of fever. Such patients may be wrongly labeled as RF. Overdiagnosis leads to undesirable stigma and exposure of patients to the rigors of an unnecessary prophylaxis. The availability of echocardiography is also limited in areas where RF is highly prevalent. The theme of arguments against the inclusion of echocardiography as a criterion is that it may pose another barrier in the diagnosis of RF. For all these reasons, it is still argued that echocardiography should not be included as a criterion for the diagnosis of RF.[30]

## INCLUSION OF ECHOCARDIOGRAPHY AS A “JONES CRITERION” – THE PROS

Even in the golden era of clinical auscultation, a number of patients with no audible murmurs in the first attack of RF developed RHD on follow-up, suggesting that carditis was missed by clinical examination.[31] Clinical skills are declining in the West,[32] and probably also in India. Murmurs may be missed even by experienced clinicians because of associated tachycardia. More importantly, it is realized that the mild valvular regurgitation is not only subclinical but, in the acute phase, even moderate mitral or aortic regurgitation may not be clinically audible. Echocardiography is more sensitive and more accurate in diagnosing valvular involvement in acute RF. Pre-existing valvular lesions can worsen due to recurrences; hence, accuracy in the diagnosis is very essential. Echocardiography not only confirms and quantifies valvular regurgitation but is also useful in establishing an alternative cause of murmur. Furthermore, echocardiography is of immense value in ruling out infective endocarditis in patients of established RHD presenting with recent onset worsening of symptoms. In areas with a high prevalence of RF, the consequences of underdiagnosis are likely to be greater than overdiagnosis. Recent analyses have suggested that subclinical carditis as a major Jones criterion influences the diagnosis of acute RF in 11-16% of patients only.[11,33] In the majority of patients, the major criterion of acute RF still remains polyarthritis, clinically overt carditis or chorea. A negative echocardiographic assessment may be useful to reassure the family and a positive finding will help to reinforce penicillin prophylaxis.

The suggested relative utility of echocardiography in various syndromes of RHD is presented in Table 3. The major confusion for the clinician in ordering an echocardiography is in patients with polyarthritis and chorea with no clinical evidence of carditis. Patients with chorea are shown to have a high incidence of clinical/subclinical carditis and the utility is not questioned.[15,21,34] The putative new syndromes of pediatric autoimmune neuropsychiatric disorder associated with streptococcal infection and post-streptococcal reactive arthritis should be diagnosed with extreme caution in Indian patients as many patients on follow-up may develop valvular lesions.

**Table 3:** Utility of echocardiography in different forms of RF/RHD

Chronic RHD	Utility not questioned
Insidious onset rheumatic carditis	Useful
Rheumatic chorea	Useful
Recurrent attacks of RF in patients without RHD	Should be useful
Recurrent attacks of RF in patients with RHD	Useful if prior echo available
Primary episode of RF	May be useful

The utility of screening all asymptomatic school children with echocardiography is not known. Recent studies have reported an unexpectedly high prevalence of RHD in Africa with the routine use of echocardiography (21.5-30.4/1000 school children).[35,36] A recent echocardiographic screening survey from India has also suggested a very high prevalence rate.[37] The authors have attributed this to subclinical carditis. The term subclinical carditis should be reserved for patients with echo-identified cardiac lesions in the clinical setting of acute RF. “Subclinical RHD” may be a preferable term for asymptomatic school children with echo-identified cardiac lesions. Because the “echo criteria for subclinical carditis” have been studied in patients with other clinical features of acute RF, it should be strictly applied to this group only. The long-term clinical outcome and the effectiveness of prophylaxis in patients with subclinical RHD should be known before this strategy is adopted in clinical practice.

## RECENT GUIDELINES

The recent Australian[38] and New Zealand[39] guidelines on RF have accepted echocardiographic subclinical carditis as a major criterion. All patients with suspected or definite RF should undergo echocardiography to identify evidence of carditis. The guidelines have also categorized severity of carditis and have recommended prophylaxis accordingly. With miniaturization of technology, echocardiography in hand-held and mobile forms may be made available in remote areas of the world. The underprivileged populations need guidelines that increase the sensitivity for acute RF to help avoid underdiagnosis. Hence, in India also we need to incorporate subclinical carditis as a major criterion as any other region with high prevalence of RF/RHD.

To conclude, the utility of echocardiography in the diagnosis of subclinical carditis is well established. However, long-term outcome of patients with subclinical carditis is less well established. It is suggested that all patients with suspected or definite RF should undergo echocardiography to identify carditis and to assess the severity of carditis. Such an approach is likely to avoid wrong diagnosis and minimize underdiagnosis.

## References

[1] Carapetis JR, Steer AC, Mulholland EK, Weber M (2005). The global burden of group A streptococcal diseases. Lancet Infect Dis.

[2] Carapetis JR (2008). Rheumatic heart disease in Asia. Circulation.

[3] WHO (2004). Expert Consultation on Rheumatic Fever and Rheumatic Heart Disease (2001: Geneva, Switzerland). Rheumatic fever and rheumatic heart disease: Report of a WHO Expert Consultation. WHO Technical Report Series.

[4] Brand A, Dollberg S, Keren A (1992). The prevalence of valvular regurgitation in children with structurally normal hearts: A color Doppler echocardiographic study. Am Heart J.

[5] Shah PM (1989). Quantitative assessment of mitral regurgitation. J Am Coll Cardiol.

[6] Lembo NJ, Dell'Italia LJ, Crawford MH, Miller JF, Richards KL, O'Rourke RA (1988). Mitral valve prolapse in patients with prior rheumatic fever. Circulation.

[7] Kostucki W, Vandenbossche JL, Friart A, Englert M (1986). Pulsed Doppler regurgitant flow patterns of normal valves. Am J Cardiol.

[8] Yoshida K, Yoshikawa J, Shakudo M, Akasaka T, Jyo Y, Takao S (1988). Color Doppler evaluation of valvular regurgitation in normal subjects. Circulation.

[9] Narula J, Chandrasekhar Y, Rahimtoola S (1999). Diagnosis of active rheumatic carditis. The echoes of change. Circulation.

[10] Saxena A (2000). Diagnosis of rheumatic fever: Current status of Jones criteria and role of echocardiography. Indian J Pediatr.

[11] Vijayalakshmi IB, Vishnuprabhu RO, Chitra N, Rajasri R, Anuradha TV (2008). The efficacy of echocardiographic criterions for the diagnosis of carditis in acute rheumatic fever. Cardiol Young.

[12] Tubridy-Clark M, Carapetis JR (2007). Subclinical carditis in rheumatic fever: A systematic review. Int J Cardiol.

[13] Caldas AM, Terreri MT, Moises VA, Silva CM, Len CA, Carvalho AC (2008). What is the true frequency of carditis in acute rheumatic fever? A prospective clinical and Doppler blind study of 56 children with up to 60 months of follow-up evaluation. Pediatr Cardiol.

[14] Rayamajhi A, Sharma D, Shakya U (2007). Clinical, laboratory and echocardiographic profile of acute rheumatic fever in Nepali children. Ann Trop Paediatr.

[15] Panamonta M, Chaikitpinyo A, Kaplan EL, Pantongwiriyakul A, Tassniyom S, Sutra S (2004). The relationship of carditis to the initial attack of Sydenham's chorea. Int J Cardiol.

[16] Karaaslan S, Demirören S, Oran B, Baysal T, Başpinar O, Uçar C (2003). Criteria for judging the improvement in subclinical rheumatic valvitis. Cardiol Young.

[17] Lanna CC, Tonelli E, Barros MV, Goulart EM, Mota CC (2003). Subclinical rheumatic valvitis: A long-term follow-up. Cardiol Young.

[18] Figueroa FE, Fernández MS, Valdés P, Wilson C, Lanas F, Carrión F (2001). Prospective comparison of clinical and echocardiographic diagnosis of rheumatic carditis: Long term follow up of patients with subclinical disease. Heart.

[19] Voss LM, Wilson NJ, Neutze JM, Whitlock RM, Ameratunga RV, Cairns LM (2001). Intravenous immunoglobulin in acute rheumatic fever: A randomized controlled trial. Circulation.

[20] Chockalingam A, Gnanavelu G, Elangovan S, Chockalingam V (2003). Current profile of acute rheumatic fever and valvulitis in southern India. J Heart Valve Dis.

[21] Elevli M, Celebi A, Tombul T, Gökalp AS (1999). Cardiac involvement in Sydenham's chorea: Clinical and Doppler echocardiographic findings. Acta Paediatr.

[22] Hoffman TM, Rhodes LA, Pyles LA, Balian AA, Neal WA, Einzig S (1997). Childhood acute rheumatic fever: A comparison of recent resurgence areas to cases in West Virginia. W V Med J.

[23] Folger GM, Hajar R, Robida A, Hajar HA (1992). Occurrence of valvar heart disease in acute rheumatic fever without evident carditis: Colour-flow Doppler identification. Br Heart J.

[24] Folger GM, Hajar R (1989). Doppler echocardiographic findings of mitral and aortic valvular regurgitation in children manifesting only rheumatic arthritis. Am J Cardiol.

[25] da Silva CH (1999). Rheumatic fever: A multicenter study in the state of Sao Paulo. Pediatric Committee - Sao Paulo Pediatric Rheumatology Society. Rev Hosp Clin Fac Med Sao Paulo.

[26] Maheu B, Costes P, Lionet P, Kamblock J, Papouin G, Mansourati J (1995). Contribution of Doppler echocardiography to the diagnosis of the first attack of acute rheumatic fever. Arch Mal Coeur Vaiss.

[27] Vasan RS, Shrivastava S, Vijayakumar M, Narang R, Lister BC, Narula J (1996). Echocardiographic evaluation of patients with acute rheumatic fever and rheumatic carditis. Circulation.

[28] Abernethy M, Bass N, Sharpe N, Grant C, Neutze J, Clarkson P (1994). Doppler echocardiography and the early diagnosis of carditis in acute rheumatic fever. Aust N Z J Med.

[29] Gerber MA, Baltimore RS, Eaton CB, Gewitz M, Rowley AH, Shulman ST (2009). Prevention of rheumatic fever and diagnosis and treatment of acute Streptococcal pharyngitis: A scientific statement from the American Heart Association Rheumatic Fever, Endocarditis, and Kawasaki Disease Committee of the Council on Cardiovascular Disease in the Young, the Interdisciplinary Council on Functional Genomics and Translational Biology, and the Interdisciplinary Council on Quality of Care and Outcomes Research: Endorsed by the American Academy of Pediatrics. Circulation.

[30] (1992). Guidelines for the diagnosis of rheumatic fever. Jones Criteria, 1992 update. Special Writing Group of the Committee on Rheumatic Fever, Endocarditis, and Kawasaki Disease of the Council on Cardiovascular Disease in the Young of the American Heart Association. JAMA.

[31] Feinstein AR, Stern EK, Spagnuolo M (1964). The prognosis of acute rheumatic fever. Am Heart J.

[32] St Clair EW, Oddone EZ, Waugh RA, Corey GR, Feussner JR (1992). Assessing housestaff diagnostic skills using a cardiology patient simulator. Ann Intern Med.

[33] Wilson NJ, Morreau J, Voss L, Stewart J, Lennon D (2005). The influence of subclinical carditis on the diagnosis of acute rheumatic fever. Heart Lung Circ.

[34] Panamonta M, Chaikitpinyo A, Auvichayapat N, Weraarchakul W, Panamonta O, Pantongwiriyakul A (2007). Evolution of valve damage in Sydenham's chorea during recurrence of rheumatic fever. Int J Cardiol.

[35] Marijon E, Ou P, Celermajer DS, Ferreira B, Mocumbi AO, Jani D (2007). Prevalence of rheumatic heart disease detected by echocardiographic screening. N Engl J Med.

[36] Carapetis JR, Hardy M, Fakakovikaetau T, Taib R, Wilkinson L, Penny DJ (2008). Evaluation of a screening protocol using auscultation and portable echocardiography to detect asymptomatic rheumatic heart disease in Tongan schoolchildren. Nat Clin Pract Cardiovasc Med.

[37] Bhaya M, Panwar RB, Beniwal R, Panwar S (2009). Echocardiographic evidence of significant regurgitation can be the sole criterion for diagnosis of probable rheumatic heart disease: Experience from a large cross-sectional survey. J Am Coll Cardiol.

[38] Carapetis JR, Brown A, Wilson NJ, Edwards KN (2007). Rheumatic Fever Guidelines Writing Group. An Australian guideline for rheumatic fever and rheumatic heart disease: An abridged outline. Med J Aust.

[39] Atatoa-Carr P, Lennon D, Wilson N (2008). New Zealand Rheumatic Fever Guidelines Writing Group. Rheumatic fever diagnosis, management, and secondary prevention: A New Zealand guideline. N Z Med J.

